# Supplementary figures and images for: Analysis of twelve genomes of the bacterium Kerstersia gyiorum from brown-throated sloths (Bradypus variegatus), the first from a non-human host
Source: PeerJ. 2024 Apr 4;12:e17206. doi: 10.7717/peerj.17206 (PMC10999152; doi:10.7717/peerj.17206)

A

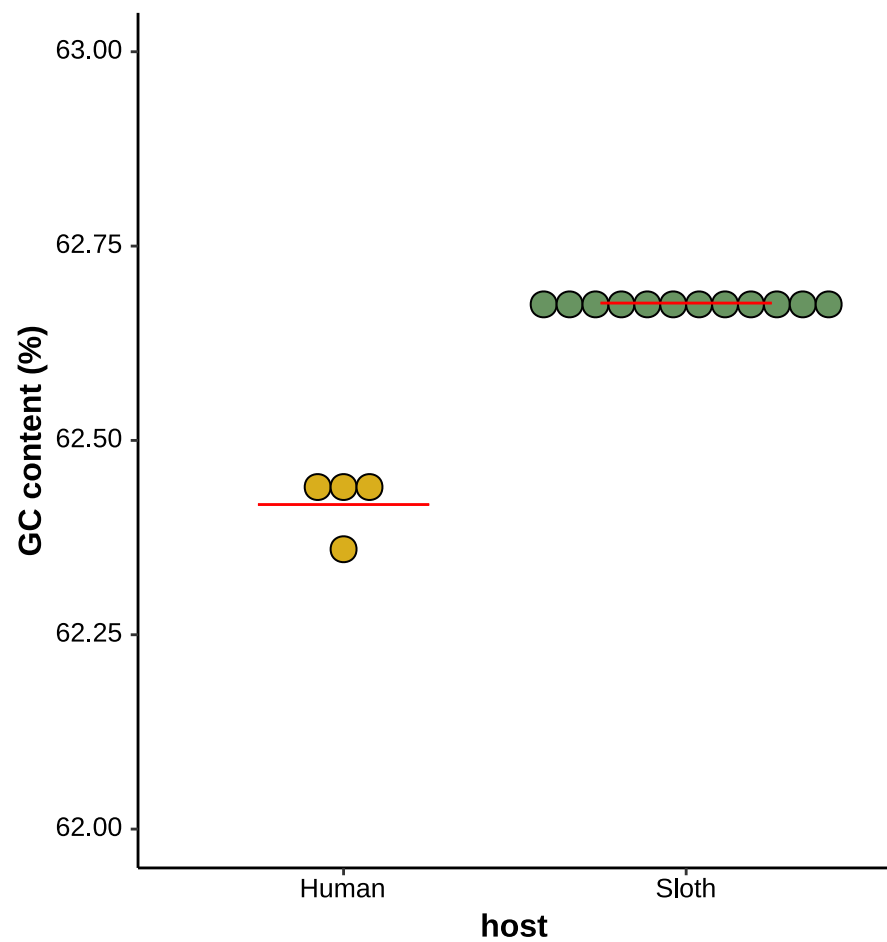

B

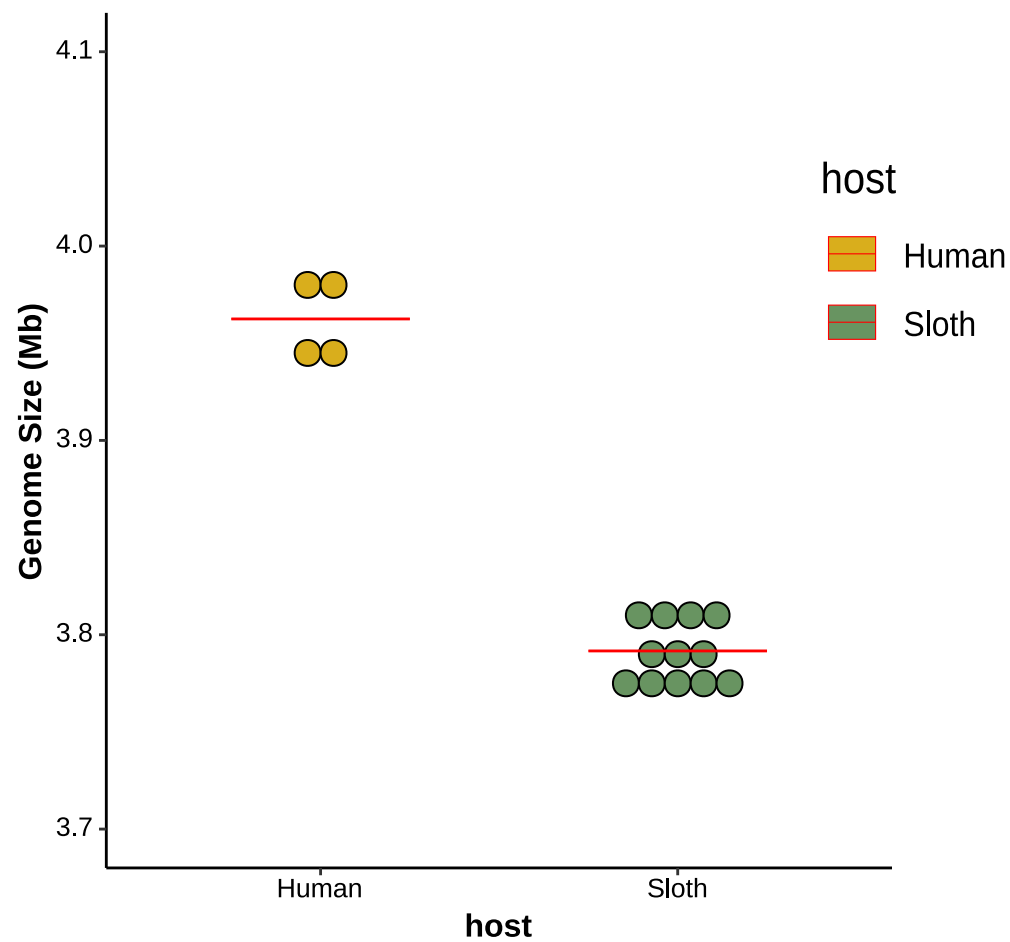

Supplement: Supplemental Information 1 [file peerj-12-17206-s001.pdf]

- GC
- DSM 16618
- CG1
- KG0001
- 3415G
- 3415D
- 2780G
- 262I
- 3324E
- 1483E
- 4201G
- 155D
- 186J
- 381J
- 186F
- RSs

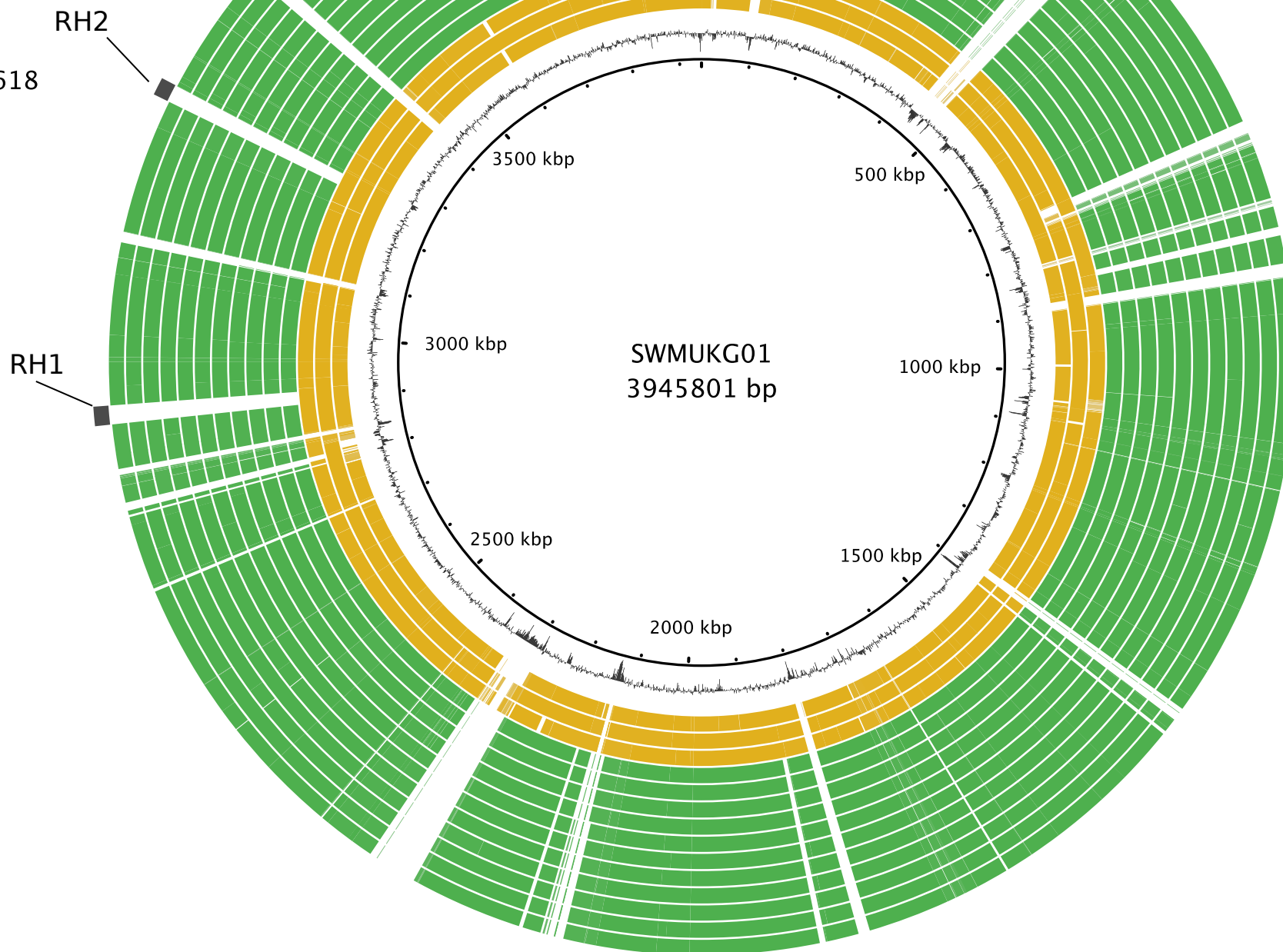

Supplement: Supplemental Information 2 — BRIG alignment of all 17 genomes of K. gyiorum reveals two genetic regions (RH01 and RH02) present in human isolates but absent in sloth isolates. [file peerj-12-17206-s002.pdf]

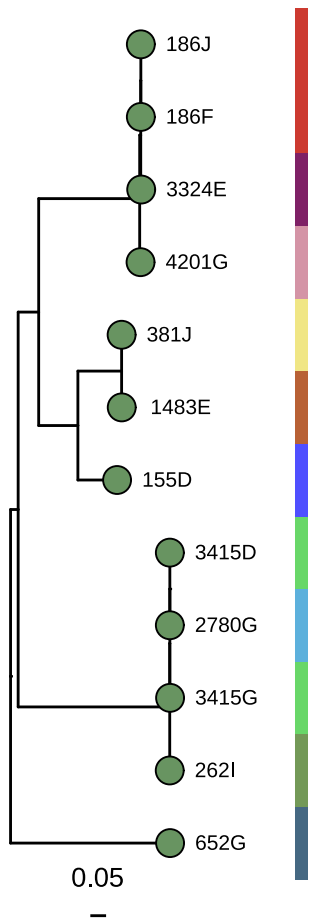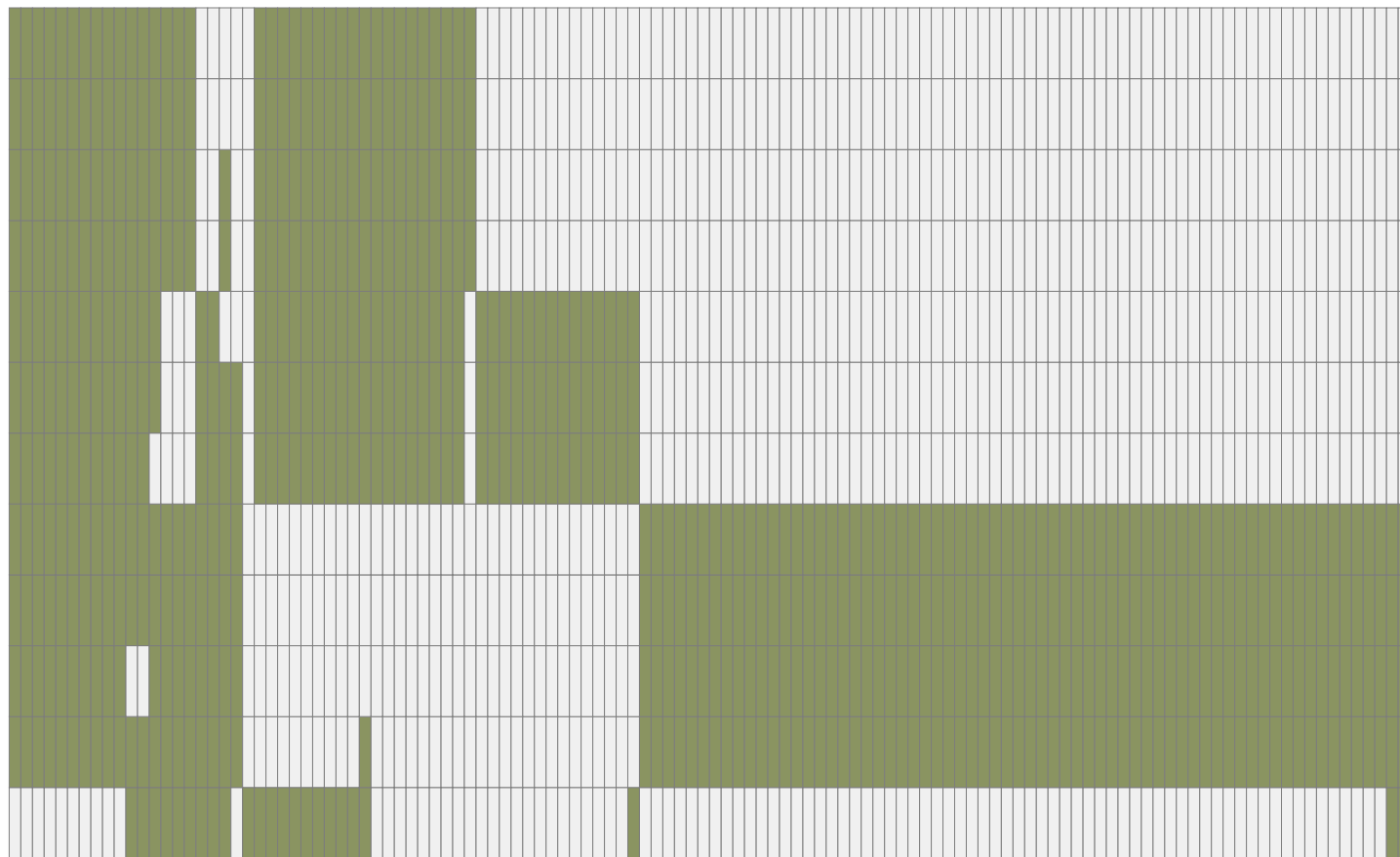

Supplement: Supplemental Information 3 [file peerj-12-17206-s003.pdf]
